# Network morphospace

**DOI:** 10.1098/rsif.2014.0881

**Published:** 2015-02-06

**Authors:** Andrea Avena-Koenigsberger, Joaquín Goñi, Ricard Solé, Olaf Sporns

**Affiliations:** 1 Department of Psychological and Brain Sciences, Indiana University, Bloomington, IN 47405-7007, USA; 2 Indiana University Network Science Institute, Indiana University, Bloomington, IN 47405, USA; 3 ICREA-Complex Systems Laboratory, Universitat Pompeu Fabra (GRIB), Dr Aiguader 80, 08003 Barcelona, Spain; 4 Institut de Biologia Evolutiva, CSIC-UPF, Pg Maritim de la Barceloneta 37, 08003 Barcelona, Spain; 5 Santa Fe Institute, 1399 Hyde Park Road, Santa Fe, NM 87501, USA

**Keywords:** evolution, complexity, graph theory, Pareto optimality, brain connectivity

## Abstract

The structure of complex networks has attracted much attention in recent years. It has been noted that many real-world examples of networked systems share a set of common architectural features. This raises important questions about their origin, for example whether such network attributes reflect common design principles or constraints imposed by selectional forces that have shaped the evolution of network topology. Is it possible to place the many patterns and forms of complex networks into a common space that reveals their relations, and what are the main rules and driving forces that determine which positions in such a space are occupied by systems that have actually evolved? We suggest that these questions can be addressed by combining concepts from two currently relatively unconnected fields. One is theoretical morphology, which has conceptualized the relations between morphological traits defined by mathematical models of biological form. The second is network science, which provides numerous quantitative tools to measure and classify different patterns of local and global network architecture across disparate types of systems. Here, we explore a new theoretical concept that lies at the intersection between both fields, the ‘network morphospace’. Defined by axes that represent specific network traits, each point within such a space represents a location occupied by networks that share a set of common ‘morphological’ characteristics related to aspects of their connectivity. Mapping a network morphospace reveals the extent to which the space is filled by existing networks, thus allowing a distinction between actual and impossible designs and highlighting the generative potential of rules and constraints that pervade the evolution of complex systems.

## 1. Introduction

A wide range of complex systems, including economic relations, the Internet, social media, ecological webs, cellular metabolism, gene regulation and brain connectivity can be represented and modelled as networks of interconnected elements [1–3]. The structure of these networks is defined by the relations among elements and interactions (nodes and edges, jointly forming the network's topology), and network function involves dynamic processes (social interactions, message traffic, chemical reactions, neuronal signalling) unfolding within this topology. While much progress has been made in characterizing features of network topology and classifying network architectures based on descriptive network metrics [4,5], numerous open questions remain. For example, it has been noted that a number of aspects of network organization, including the existence of modules [6] and small-world attributes [7], are encountered across many different complex systems. However, it remains unclear whether these commonalities reflect shared constraints on network function that limit the range over which design parameters of real-world networks can vary, or whether the space of possible network architectures is much larger than current instantiations suggest.

The idea of distinguishing between what exists, what is possible but does not exist and what is impossible has been explored in the study of the configuration and shape of biological forms, and it has helped to shed light on their evolutionary origins. As François Jacob [8] pointed out, evolution proceeds, to a large extent, through tinkering: it is obliged to re-use what is available, and this necessity forces many living structures to evolve by combining or incrementally modifying existing forms and patterns. In this context, it has been shown that reuse (as it occurs with gene duplication) can explain some global features exhibited by different kinds of natural and man-made networks [9,10]. But tinkering is only part of the whole story. Major innovations can emerge from time to time as the fundamental logic of the processes involved allows major transitions to occur [11]. Moreover, strong dynamic constraints operate on top of evolutionary processes, canalizing the potential paths to be followed by natural forms [12]. Canalization not only increases the likelihood of the emergence of some forms over others, but it also excludes from actual existence a large set of possible forms. The widespread presence of convergent designs [13] is strong evidence that the repertoire of possible forms is limited—examples are patterns of morphological organization that recur across many different classes of organisms (e.g. eyes) [14]. Such a view implies that the repertoire of possible structures [15,16] or even dynamical patterns [17] might be more limited than we would expect.

**Figure 1.**
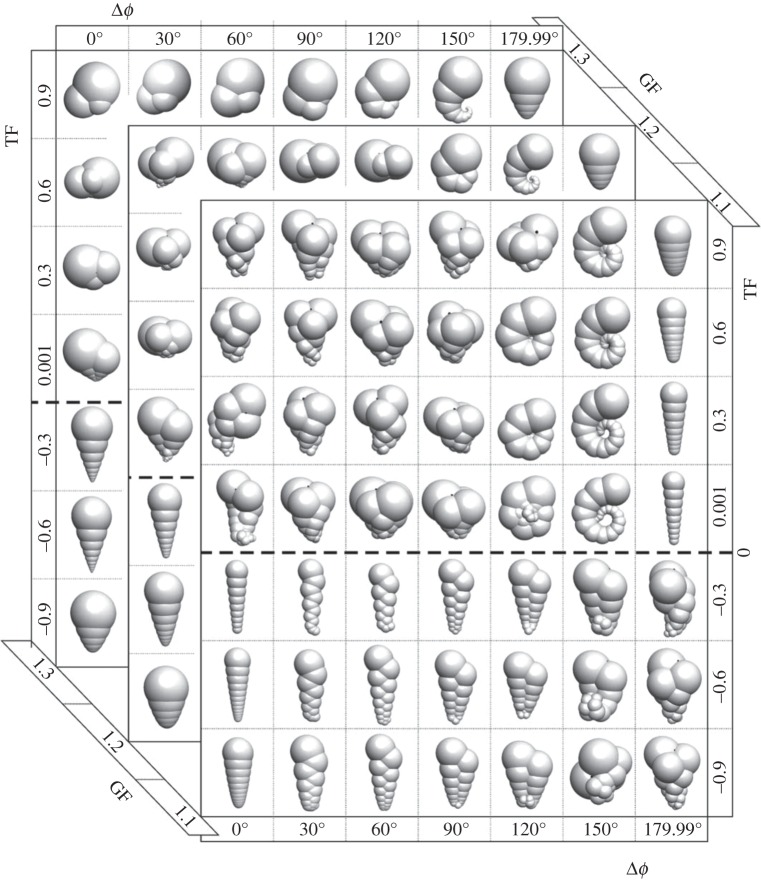
Theoretical morphospaces allow organization of morphological complexity for a given group of organisms (usually focusing on some external, anatomical traits) within a limited phenotypic space. Here, we show a three-dimensional theoretical foraminiferal morphospace. The potential repertoire of Foraminifera shells is generated by a three-parameter model of form, whose parameters are: Δ*φ*, deviation angle; translation factor, TF; growth factor, GF. Adapted with permission from reference [21].

Quantifying similarities and regularities among evolving biological forms would be facilitated with the definition of a phenotypic space within which different forms can be placed and related to one another. An important development in this regard was the formulation of the concept of ‘theoretical morphospace’ [18]. Through this approach, evolutionary biologists have been able to establish mathematical models of form that describe the entire spectrum of physically possible forms available for a given taxonomic group. Classical examples of this approach are David Raup's studies of coiled shells [19,20] (figure 1) and Karl Niklas' studies of plant evolution [22–24]. Both, Raup and Niklas provided simple mathematical models that generate the entire spectrum of coiled forms and ancient land plant variants, respectively [25]. Analysis of these mathematically generated forms revealed that variants that have existed in nature are confined to discrete regions within the total spectrum of possible forms. Additionally, Niklas showed that the number and occupation of optimal phenotypes increases with biological complexity, which he defined as the number of tasks an organism has to perform simultaneously in order to grow, survive and reproduce [24].

These attempts to conceptualize and map morphological complexity have so far not made much contact with efforts to characterize patterns of network complexity. Some classification schemes based on network structure have been proposed, capable of identifying inherent structural differences between distinct networks classes [26–28], such as social networks, information and technological networks, and biological networks. In this paper, we review and expand on recent work that combines the concept of theoretical morphospace with emerging concepts from the study of complex networks. We begin by briefly outlining some fundamental points about modern approaches to characterize morphological complexity and to measure structural attributes of networks. We then introduce the concept of ‘network morphospace’ that allows not only to build maps of network architectures, but also to delineate regions within this space that are encountered in the real world, thus allowing a distinction between designs that actually exist from others that do not exist, but may be either possible or impossible alternatives. We briefly describe the steps necessary to analyse network morphospaces and discuss the kind of information that such analysis contributes to the understanding of complex networks. Finally, we turn to a survey of studies that have begun to use the morphospace approach in order to identify and classify important characteristics of network structure.

## 2. Theoretical foundations of network morphospace

### 2.1. Morphological complexity

The work of many comparative anatomists, embryologists and evolutionists has contributed to the emergence of theoretical morphology as a discipline that studies the evolution of biological forms. A crucial concept derived from the work of the geneticist Sewall Wright [29], who proposed that, in theory, it is possible to construct a space of all possible genetic combinations associated with living organisms. Wright's major insight was that the majority of the possible genetic combinations would not actually exist in nature because they have zero fitness, whereas only a small fraction of possible combinations have fitness greater than zero and thus, are potentially present in nature. Wright proposed that these genetic relationships could be expressed as spatial or geometric relationships by constructing an *N*-dimensional space, with *N* − 1 dimensions corresponding to a set of genetic traits, plus one more dimension corresponding to fitness. This defines a fitness landscape, where the elevation of the terrain represents the degree of fitness. In this landscape, possible combinations of genetic traits that are actually observed in nature are located on peaks or elevated slopes (depending on their level of fitness), whereas possible but non-existent combinations of genetic traits are found on a flat plane of zero fitness.

Equivalent to the concept of fitness landscape for genetic traits, an adaptive landscape describes the different possible morphologies that are available in nature. In this case, *N* − 1 dimensions correspond to a set of morphological traits and, the *N*th dimension indicates the degree of adaptation. The degree of adaptation of the possible morphological traits is determined by how well such morphological traits function in nature [24].

A related concept, the *theoretical morphospace*, has been defined by George McGhee [30] as ‘*N*-dimensional geometric hyperspaces produced by systematically varying the parameter values of a geometric model of form’. In other words, the dimensions of a theoretical morphospace are the parameters of geometric or mathematical models of form, and different points along each dimension correspond to values of model parameters that specify specific forms. Then, an extra dimension can be added to indicate the frequency of occurrence of the model forms in nature. Hence, within the morphospace, it is possible to determine which forms have been produced in nature and which have not. Non-existing forms are not necessarily non-adaptive or have zero fitness; it could be that the process of evolution has simply not produced them. It is worth noting that not having observed a form in nature is insufficient evidence to assert that it has never existed.

The most important feature of theoretical morphospaces is that the dimensions are defined independently of any measurement data of existing form; thus, all possible forms, existent and non-existent, are represented within this space by varying the parameter values of a model of form. Interestingly, the measurement-independence property of the dimensions of a theoretical morphospace allows for the addition of arbitrary dimensions (as long as more parameters can be included in the mathematical model); in particular, the dimension of ‘degree of adaptation’ could be mapped onto a theoretical morphospace, thus converging to the adaptive landscape. This convergence makes theoretical morphospace analysis a very powerful tool for analysing the functional significance, the adaptive value or the fitness of existing and non-existing morphologies.

### 2.2. Network complexity

Network structure refers to topological properties that are defined by the way in which network nodes are connected to each other. The topology of a network with *N* nodes is completely defined by an *N* × *N* adjacency matrix *A*, where each element *a_ij_* of the matrix is non-zero if nodes *i* and *j* are connected, and zero otherwise. While network topology discounts metric or spatial relations among network elements, many real-world networks are spatially embedded [31,32]. Examples are brain networks [33,34], transportation networks [35,36], the internet and electrical circuits [34,37–39], and even social networks [40,41]. Spatial embedding may place important constraints on network topology [31,32]; therefore, the study of network topological features often takes into account the spatial relations.

A number of fundamental topological properties, such as hierarchical and modular organization, heterogeneous degree distributions or short characteristic path length are common among most real-world networks [42,43]. Thus, it appears that the universe of all possible network architectures is much less diverse than might be expected. The possible causes of the regularities observed across different real-world systems continue to be a matter of debate. One view is that there are strong constraints on the structure of networks imposed by fundamental laws of a mathematical nature [44]. That is, the set of attainable structures within the universe of all possible networks is subjected to mathematical constraints, and hence real-world networks occupy a very small subset of this universe. Under such a view, a number of structural features found in most real-world networks, such as network motifs [45,46] and modularity [47–49], may be viewed as evolutionary ‘spandrels’ [46], that is, they have not been actively selected for, but instead, they are the by-product of the underlying generative rules, with little connection to functional constraints. An alternative view suggests that the emergence of common features in nature may arise from a combination of growth mechanisms and the interplay of network dynamics with selection processes [50]. Interestingly, in spite of the diverse nature of the dynamical processes taking place on distinct network classes, most of these processes are associated with the transmission and/or processing of information, driven by the pairwise interactions among the system's components [51]. Thus, it seems plausible to hypothesize that the convergence of many networks towards a common set of properties is driven by optimization processes that favour (directly or indirectly) the transmission and/or processing of information.

Clearly, beyond the goal of defining classification schemes for complex networks, there are fundamental questions that are still unanswered. What are the factors that shape network structure? How do the structural properties of a network arise in the course of network growth and evolution, and how do different structural properties interact with one another? Are common features of complex systems a result of common selection pressures or do they emerge as a result of structural/functional constraints? Considering systems that evolve and whose structure changes with time, what are the possible organizational changes that such networks can support?

### 2.3. Defining network morphospaces

One approach to address these questions is to map networks to *N*-dimensional spaces whose axes are defined by specific network attributes, and then analyse their distribution within this ‘network theoretical morphospace’ (henceforth ‘morphospace’). However, drawing an analogy between physical forms and network structure is not straightforward: while theoretical morphology is able to model the essence of many physical forms with mathematical or geometrical relationships between morphological traits, the complex topology of most real-world networks makes it hard to define mathematical models that completely specify a network's structure as a function of a set of independent structural traits. One way forward builds on the many analytic measures and tools used to determine the statistical properties of networks [42,52,53]. We may take a set of network statistical properties to represent structural traits, and the combination of these traits can then be associated with characteristic network topologies. These network measures, interpreted as structural traits, form the dimensions of a morphospace, where combinations of traits are associated with distinct ‘network forms’.

**Figure 2.**
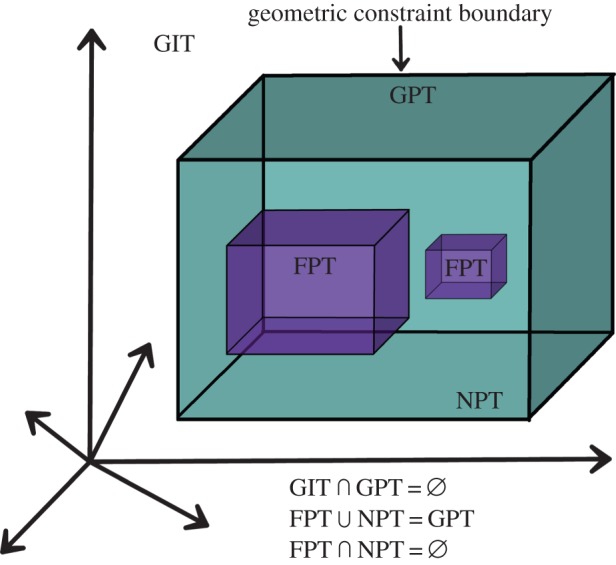
Illustration of a theoretical morphospace: an *N*-dimensional hyperspace where each dimension (or axis) represents a network topological trait. Extrinsic constraints define the boundaries between the GIT region and the GPT region and the boundary between the *functionally possible topology* (FPT) region and the *non-functionally possible topology* (NPT) region. Note that the spatial distribution of the distinct regions may not be contiguous, as long as the non-overlapping set properties are preserved. Modified from McGhee [30].

One of the most important objectives of morphospace analysis is to delineate the boundaries that define the domains of actual, possible and impossible forms (figure 2). In network morphospaces, these different regions are analogous to those described by McGhee in the context of biological morphologies (in fact, we use a nomenclature based on the one proposed by McGhee [30] to refer to these distinct regions).

The first subdivision of the space is defined by extrinsic constraints that are imposed by geometrical laws; these constraints define the region of geometrically possible topologies (GPT) and the region of geometrically impossible topologies (GIT) whose intersection is empty (i.e. 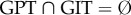). Examples of GIT are a network with five nodes, all connected to exactly three neighbours, or a connected network with an average path length of zero. Within the GPT region, there are two subregions defined by another class of extrinsic constraints, called functionality constraints, which separate functional possible topologies (FPT) from non-functional possible topologies (NPT). Functionality constraints are imposed by physical or geometrical laws that determine whether the system represented by the network can actually function or not. As an example, consider a morphospace of protein interaction networks. While it is topologically possible to define a network in which all nodes (proteins) interact with each other (a clique), the laws of physics prohibit many of these interactions taking place in nature; therefore, a fully connected protein interaction network is contained in the NPT region of the protein interaction morphospace. Note that ‘functionality’ is dependent on the nature of the physical or biological system under study. That is not to say that physical laws behave differently for different systems, but some physical laws are only relevant for the analysis of certain systems.

Finally, there are intrinsic constraints that are imposed by the properties of the specific system represented by the network. For example, consider a morphospace of human brain networks. A fundamental constraint on human brain networks is that they are embedded in space. Spatial embedding is closely tied to incontrovertible limits on brain volume, conduction delays and metabolic energy consumption [33,54]. Global limits on brain volume impose strong constraints on the volume of grey matter (neurons or nodes) and white matter (connections or edges). Thus, resource limitations impose constraints on the density, length and volume of neuronal connections, hence separating biologically feasible and unfeasible networks within the morphospace. Note that intrinsic constraints can be imposed on all regions of the morphospace, but most importantly, by imposing intrinsic constraints during the simulation of networks, one can restrict the space of possible networks within a morphospace to those that are not only topologically and functionally possible, but also biologically feasible. This has the advantage of reducing the search space and guaranteeing that all the simulated networks comply with the set of intrinsic constraints that are required for the system to be generated and maintained.

### 2.4. Constructing network morphospace

**Figure 3.**
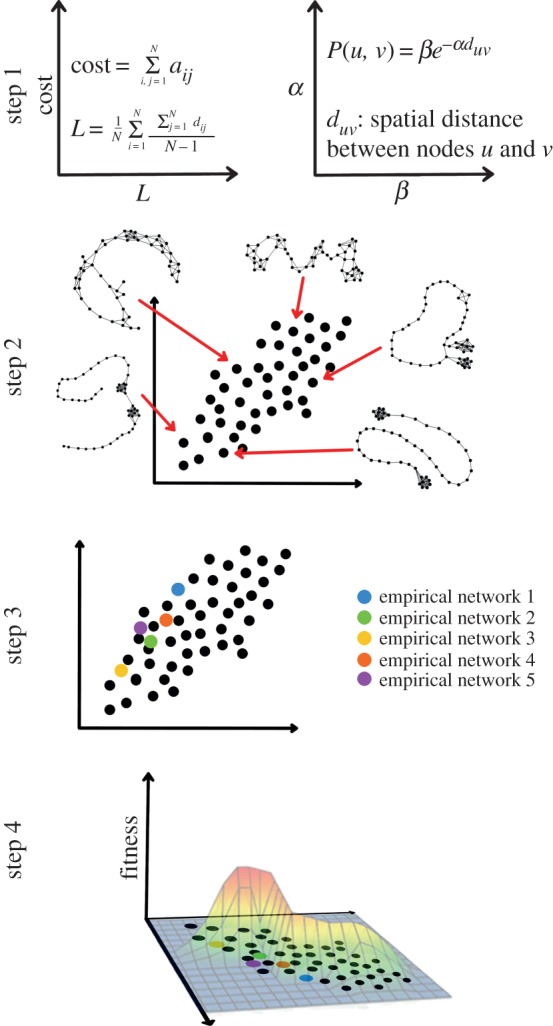
Four steps to conduct a morphospace analysis. Step 1: defining the dimensions of a morphospace. Dimensions are given by the number of structural traits considered in the model; structural traits can be measured directly from the network's adjacency matrix (e.g. connection cost and characteristic path length) or given by the parameters of a growth model (e.g. the parameters of a spatial-growth model [55]). Step 2: generating network topologies that correspond to the distinct combinations of structural trait values. Step 3: placing empirical networks within the morphospace by measuring the pre-selected set of structural traits. Step 4: morphospace analysis. This step aims to answer the question of why real networks are located in particular regions of the morphospace, and not in other regions that are both possible and functional.

How do we construct a morphospace such that it aids in the understanding and analysis of actual network structure? Let us assume that we are interested in characterizing the common structural properties of a certain type of network (e.g. protein interaction networks, electrical circuits or social networks) and, furthermore, that we are interested in studying the possible factors shaping the structure of such networks. We can conduct a morphospace analysis to address these questions by following a series of four steps (figure 3).

#### 2.4.1. Step one: defining the dimensions of the morphospace

Constructing a morphospace involves a choice of structural features to analyse and a mathematical model to measure them. Of course, these decisions are made with respect to the hypothesis under consideration; a more detailed characterization may require a higher-dimensional morphospace, which may be difficult to analyse and visualize. There are two ways in which the dimensions of a morphospace can be defined. One way is to use a set of structural traits as the morphospace dimensions, as illustrated in the top left axes of step 1, in figure 3. These traits are defined by the elements of the network's adjacency matrix, and a mathematical model is required to measure them. Examples of structural traits are average degree, characteristic path length, measures of modularity and hierarchy, among others. A second way to construct a morphospace is to use a generative model of network growth. In this case, the dimensions of the morphospace are the parameters of the growth model. Examples of such models are the Barabasi–Albert preferential attachment model [56] or the spatial-growth model proposed in reference [55], illustrated in the top right axes of step 1, in figure 3. It is worth mentioning that network growth models generally are not deterministic; that is, for a fixed set of parameters, a growth model can generate different instances of networks.

#### 2.4.2. Step two: generating network topologies

If the morphospace is defined by a model of network growth, then filling the morphospace involves ‘growing networks’ while systematically varying the model parameters. If the morphospace is defined by a set of structural traits, then generating network topologies corresponding to all the possible values of structural traits is a non-trivial problem. This is because the structural traits are functions of the elements of the adjacency matrix, but the relationship between structural traits and adjacency matrices is not one-to-one—rather the mapping between traits and networks is degenerate in that different adjacency matrices can yield the same combination of structural traits. This degeneracy implies that the mapping of matrices onto traits cannot be inverted (given a set of structural traits, one cannot uniquely specify the matrix from which it originated). Hence, there is no simple procedure that can generate candidate networks for all possible combinations of structural parameters. A number of approaches have been proposed to address this problem; most of them consist of the implementation of optimization techniques that explore the morphospace by generating candidate networks and evolving them to attain specific structural trait values.

#### 2.4.3. Step three: placing empirical networks into the morphospace

Empirical networks are placed within the morphospace by measuring the pre-selected set of structural traits of interest, which can be derived either from their adjacency matrix or by determining the parameter values of the network's growth model. There is one consideration that requires special attention: it is well known that graph measures (i.e. structural traits) depend on the number of nodes and edges (i.e. network size and density) in a way that is specific for the type of network topology [57]. Thus, a comparison of the structural traits of networks that vary in size cannot be performed directly and normalization or scaling of measures is required. There are various approaches to correct for size dependence, although some of these approaches may introduce statistical biases. In general, the comparison of networks of different size and density is still an open problem [57].

#### 2.4.4. Step four: morphospace analysis

The completion of steps two and three enables the identification of the boundaries and regions of a morphospace where actual, possible and impossible network topologies are located. Furthermore, the functionality and fitness of all networks located within the GPT region can be evaluated, for example, in terms of other structural traits that are relevant for the performance of a given system, in one or several tasks. Functional analysis aims to address the question of why real-world networks are located in particular regions of the morphospace, and not in other regions that are both possible and feasible. We mentioned previously that it is possible to add fitness as a dimension of the morphospace, transforming the morphospace into an adaptive landscape.

## 3. Studies exploring network morphospaces

In this section, we survey some recent examples of studies that have examined network morphospaces in different domains. We focus particularly on describing the different exploratory strategies that have been proposed in order to sample from the entire spectrum of possible topologies available within a morphospace. We start by exemplifying how to extend a network hyperspace into a morphospace analysis, for example in the context of network evolution. We then turn to exploratory strategies within morphospaces whose axes are defined in terms of structural traits. Finally, we review morphospaces whose axes are defined by the parameters of a model of network growth.

### 3.1. Network evolution and morphospace analysis

There have been several attempts towards characterizing and classifying complex networks [58,59]. It is not within the scope of this paper to review these methods, nonetheless, it is worth pointing out that many network classification schemes can be extended into the morphospace framework, providing additional information. For example, one of the initial attempts to characterize networks is based on the classification of *network motifs* [45]. Motifs may be thought to represent the basic ‘building blocks’ that constitute a network and thus, the frequency with which they occur may be informative of which specific connectivity patterns have been selected for network function. A motif morphospace, where motif frequencies form the axis of the space, can provide information about the functionality of distinct classes of networks, reflecting which motif distributions are easier to generate and which are impossible to generate, and possibly identifying distinct motif frequencies that are correlated in simulated and/or real-world networks.

Although the formal concept of network morphospace is only a recent addition to the field, network hyperspaces (or phase-spaces) have been widely used to characterize and classify networks, and to study network evolution. For example, a *clustering signature* hyperspace has been defined to characterize real-world-directed networks in terms of four components that indicate how nodes are connected to their neighbourhood [60]. Networks can also be classified by analysing and comparing the trajectories followed by evolving networks within a hyperspace [61], for example, when networks are subjected to some kind of selection pressure [62–67]. This approach has been particularly useful to identify connectivity patterns that optimize a trade-off between two antagonistic structural properties, such as the trade-off between network cost and efficiency [63] or the trade-off between network cost and synchronization [64,65].

Like network morphospaces, network hyperspaces are *N*-dimensional spaces whose axes represent specific network measures. However, it is worth making a distinction between a comprehensive morphospace analysis and the more heuristic use of hyperspaces to study networks. A key difference is that a hyperspace is simply defined by specifying the axes of the space. Conversely, a full morphospace analysis focuses on characterizing the extent and distribution of the distinct subregions of the space (i.e. GPT, FPT; figure 2), while associating specific aspects of network topology with these regions. As we have argued previously, one of the most powerful analytic aspects of theoretical morphospaces is their ability to explore all network topologies available for a particular system, enabling the identification of functional constraints or selection forces that disallow their existence.

The observation that most complex networks display common topological attributes motivated a study [68] to explore the possibility that such commonalities stem from the presence of optimization processes. The study used an optimization algorithm that evolved networks to simultaneously optimize two relevant (and antagonistic) aspects of network performance: the cost of building/maintaining connections and the communication speed (*ρ*) among nodes. A parameter *λ* (0 ≤ *λ* ≤ 1) controlled the importance given to cost and communication speed when searching for optimal networks (by means of the parametric linear equation *F*(*λ*) = *λ*(cos*t*) + (1 − *λ*)*ρ*); providing distinct optimal solutions, depending on the value of *λ*. The results of this study showed that many aspects observed in complex networks can emerge from distinct solutions that optimize a trade-off between two objectives. Viewed from the perspective of a morphospace analysis, the cost-communication features of any network define its coordinates within a two-dimensional morphospace, whose axes are network cost and communication speed. The optimization algorithm from [68] operates within this morphospace by implementing an exploratory strategy: every value of *λ* used in the optimization is associated with a trajectory or sequence of evolved networks. The set of all trajectories corresponding to all values of *λ* defines the region of morphospace that is accessible through this exploratory strategy; in this way, the distinct optimal solutions (topologies) can be associated with different regions of the explored morphospace. Interestingly, locating real-world networks within this morphospace could possibly allow us to conjecture whether connection cost or communication speed have a stronger influence in driving network architecture.

The previous analysis shows that, in general, the process of optimizing or evolving a set of network topological or dynamical features (such as in references [62–67]) can be approached and extended into a morphospace analysis. A sequence of evolved networks that lead to an optimal or nearly optimal network (according to a given performance measure) represents a trajectory within the GPT region of a morphospace. The advantage of studying optimality within the morphospace framework is that it allows us to extract more information about the accessibility to distinct solutions (i.e. possible versus impossible solutions, functional versus non-functional solutions) in addition to studying the behaviour of the trajectories that lead to optimality. Most important, this framework can be used to test hypotheses about mechanisms underlying network organization, such as selection pressures and functional/structural constraints.

### 3.2. Structural constraints on network organization

Understanding the relationship between a network's topology and its ability to evolve is fundamental to understanding the principles that drive network organization; in this respect, it is particularly interesting to identify the evolutionary constraints imposed by a network's topology and furthermore, by a network's function.

It is well known now that in spite of the diverse evolutionary processes that drive the formation of different systems, they all converge towards heterogeneous, modular architectures that show a balance between order and randomness [69]. Solé and Valverde [44] addressed the question of whether such convergence is the product of selection pressures or the product of fundamental constraints on the available network topologies. Two information-based functions were developed to study the heterogeneity of the node degrees (*H*, corresponding to the entropy of the degree distribution), and the correlations between connected nodes' degrees (*H*_c_, measured by the entropy of the conditional probability of observing a node with *k* connections, provided that the node at the other end of the chosen connection has *k*′ connections). These measures or structural traits define the axes of a morphospace in which several real-world and theoretical model networks were located. Interestingly, all networks were located extremely close to the identity line *H*_c_ = *H*, a behaviour that results when node degrees are statistically independent; that is, node degrees are not highly correlated, or at least not more than what would be expected by chance. Given the mathematical formulation of *H*_c_ and *H*, it is the case that *H*_c_ ≤ *H*, therefore, the region of the morphospace, where *H*_c_ > *H* is contained in the GIT region. In order to determine the principles preventing networks from occupying the region *H*_c_ < *H*, simulated annealing was used to rewire candidate random networks with the objective of minimizing the distance between such networks and randomly sampled points (*H*,*H*_c_). The surprising result of this exploration is that the distance is effectively minimized only for points sampled along the line *H* = *H*_c_, which is the same region where real-world and canonical networks are located. In addition, the optimal networks found by the algorithm displayed scale-free degree distributions, a feature observed in several real-world networks [42].

These results suggest that there are fundamental constraints imposed on network structure that make most of the morphospace unattainable. Regarding this conjecture, there are two important considerations to point out. First, results in reference [44] may be limited owing to the exploratory strategy used. A scenario to consider is that the morphospace landscape is such that it is very unlikely to find a sequence of structural changes that transform random topologies into topologies that do not exhibit heterogeneous degree distributions; we can think of a landscape having a canal that directs random networks towards the region of morphospace occupied by networks with heterogeneous degree distributions whose node degrees are statistically independent. Perhaps there are alternative topologies that occupy regions where the landscape is easier to travel in several directions. The second consideration is regarding the kind of constraints that make most of the morphospace unattainable. Given that *H* is computed as the entropy of the degree distribution, it must be noted that there is a finite number of degree distributions that a network can attain, given a fixed number of nodes and connections. As an example, let us consider the case in which the entropy of the degree distribution takes its maximum value, that is, when there is a uniform probability of observing a node with 1,2,3, … , *n* connections. Now, a hand-made drawing may suffice to show that given a fixed number of nodes, there are many degree distributions that are impossible to construct, including the ones that yield the maximum entropy (to illustrate this, try drawing a five node network, with a degree probability distribution that is uniform). Thus, the entropy values that can be associated with a network are restricted; as a consequence of these extrinsic constraints imposed on all network architectures, the domain of the GIT region spans the majority of the morphospace.

**Figure 4.**
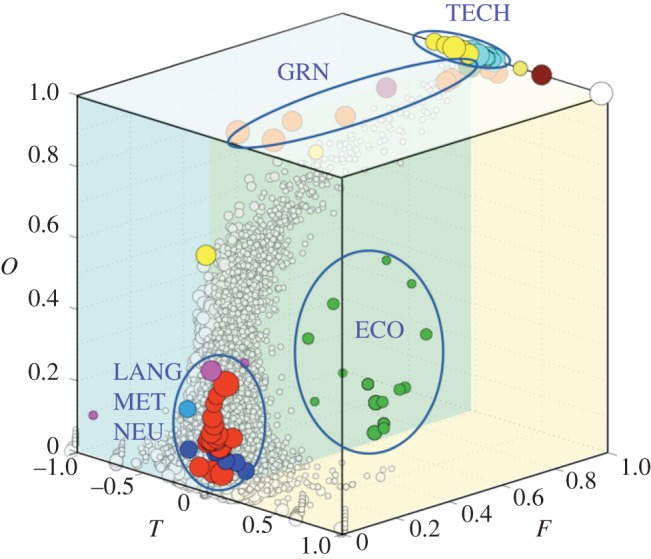
Hierarchy morphospace. The axes of this three-dimensional morphospace are given by treeness (*T*), feed-forwardness (*F*) and orderability (*O*). Coordinates of 125 real-world networks are indicated with filled circles, coloured according to network type (TECH, electronic circuits; GRN, GRNs; ECO, food webs; LANG, world corpora; MET, metabolisms; NEU, neuronal); four clusters can be identified, according to network's location within the morphospace. Non-coloured spheres represent ensemble of random networks of various sizes and degree distributions. Reproduced with permission from reference [70].

A recent study [70] employed a morphospace approach to examine the hierarchical features of complex networks and understand the forces that shape hierarchical directed networks. To characterize and quantify network hierarchy, three measures were defined: (i) *treeness*, a measure of how pyramidal the structure is; (ii) *feed-forwardness*, a measure of the impact of cyclic modules on the structure of a network, based on their position within the pyramidal structure; (iii) *orderability*, a measure of how orderable the network is, based on the fraction of nodes that does not belong to a cycle. Within the three-dimensional morphospace defined by these measures, an ensemble of random networks with homogeneous and heterogeneous degree distributions and 125 real-world networks of natural and artificial systems were located (figure 4). Surprisingly, in spite that four clusters of real-world networks with particular hierarchical features are distinguishable, almost all networks were found to occupy the same region occupied by the random ensemble. Because random networks are not considered to be optimally designed towards any structural trait, it is assumed that no selection pressures have restricted their occupation within the morphospace. Thus, the fact that real-world networks and random networks are located within the same region suggests that hierarchical order may be a by-product of random fluctuations that possibly emerge from selection for other structural traits, such as cost minimization or robustness against node and/or connection failure.

In order to inquire whether the regions unoccupied by real-world and random networks are constrained by evolutionary selection pressures or belong to the GIT region, an exploratory strategy similar to the one used in reference [44] was employed: a set of random networks were evolved to minimize the distance between them and a set of target point taken from a gridded partition of the morphospace. The results indicate that regions occupied by random and real-world networks are easily accessed, that is, most trajectories followed by evolved networks end up in this highly occupied region. However, other regions of the morphospace are not accessible by means of this exploratory strategy. These results are in agreement with the hypothesis that the hierarchical features of most networks are not selected for, but actually emerge spontaneously, driven by a (constrained) trend for networks to evolve towards a specific region of the morphospace.

### 3.3. Trade-offs and selection pressures in network evolution

Trade-off situations are commonly encountered in theories of natural selection. Organisms that perform a range of tasks cannot be optimal at all tasks; therefore, the interplay between multiple traits that contribute to the fitness of an organism face fundamental trade-offs that restrict the range of possible trait values that organisms can adopt. Models that implement network evolution through selection processes may optimize a fitness (or energy) function *F*(*s*) that combines a set of desirable traits *s*. In this way, weights can be associated with each trait, determining how important such traits are with respect to each other. However, there are some limitations to this approach. First, one has to define the form of the fitness function and determine how the different traits (or objectives) interact with each other. For example, the weights associated with each trait can be varied in order to find distinct optimal solutions for the different trade-off combinations. Second, to define the fitness function, one has to know whether the objectives are antagonistic or not. Third, the problem becomes more difficult as the number of objectives increases because of all the possible combinations in which traits can interact with each other. In a recent study, Shoval *et al.* [71] applied the concept of Pareto-optimality within trait space (as opposed to performance space, in which it is usually applied), bypassing these limitations and interestingly, showed that when organisms need to perform different tasks, the best trade-off solutions occupy low dimensional regions within the morphospace.

Indeed, as suggested long ago by Wright [29], and later by McGhee [30], the morphospace regions that are, or have been, occupied by existing morphologies (topologies) are very sparse; in other words, most empirical morphospaces are mostly empty. The challenge now is to explore the entire set of geometrically and functionally viable morphologies (topologies) available for a given system, and understand why only a subset have been observed in nature. Two recent studies attempted to address this question from the perspective of communication efficiency in complex networks [72] and subsequently within the domain of anatomical brain networks [73].

**Figure 5.**
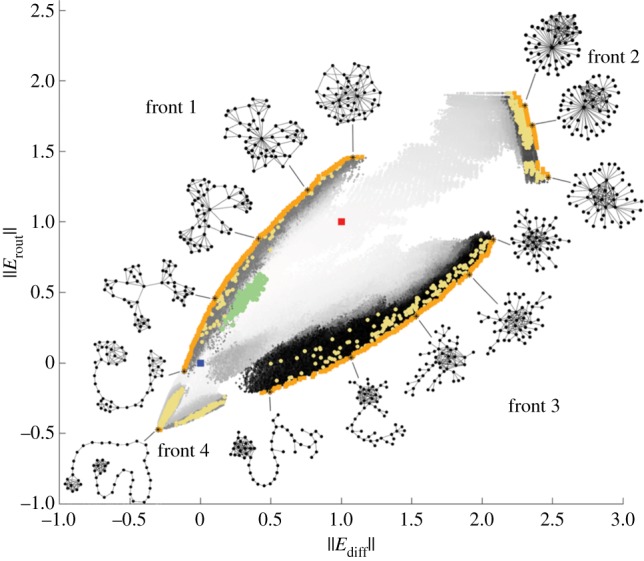
Communication-efficiency morphospace. Every point represents a network generated by the optimization algorithm. The regions explored by four independent simulations are shown. Each simulation corresponds to a different combination of objective functions which drive a population of networks towards four quadrants or the morphospace. Green points indicate the location of the initial population; blue and red points indicate the location of the lattice and random networks, respectively; orange points show the location of the final population of networks generated by each simulation. A sample of networks selected from the final population of networks shows that distinct topologies can be associated with different morphospace regions. Reproduced with permission from reference [72].

In [72], a two-dimensional communication-efficiency morphospace was defined in order to study and characterize specific aspects of network structure that favour efficient communication within a network. Two kinds of communication schemes were considered in this study: *routing communication* (originally defined in reference [74]), which takes place through the shortest paths between nodes, requiring global knowledge of the network structure in order to find such path; *diffusion communication*, which is a diffusion-like process through which information can propagate (as a random walk) in the absence of global knowledge about the network structure. Two structural measures, *E*_rout_ and *E*_diff_ quantify a network's efficiency to communicate through routing and diffusion processes respectively [72] and define the axes of a morphospace (figure 5). A multi-objective optimization algorithm was implemented as an exploratory strategy that drives a population of networks towards four quadrants of the two-dimensional space. However, instead of defining a single global objective function (such as in references [44,68,70]), network selection was carried out according to Pareto-optimality. In general, a solution is said to be Pareto-optimal if an improvement of any single objective cannot be achieved without making some other objective worse [75]. In the context of a population of networks that are evaluated by multiple objective functions, a network *Γ* belongs to the Pareto-front set if and only if (i) *Γ* is not worse than any other network within the population, with respect to all objectives and (ii) *Γ* is strictly better than any other network in the population, with respect to at least one objective [76]. The exploration of the efficiency-communication morphospace (figure 5) reveals that the GPT region is severely restricted, thus it is not possible to generate networks with arbitrary values of *E*_rout_ and *E*_diff_. While most networks are located near the line *E*_rout_ = *E*_dif_, it is possible to identify characteristic topologies that deviate from this behaviour or that favour one communication scheme over the other. Nonetheless, the strong linear dependency between *E*_diff_ and *E*_rout_ suggests a trade-off situation, where selection pressures might drive systems to occupy a low dimensional region (a line) within the communication-efficiency morphospace.

**Figure 6.**
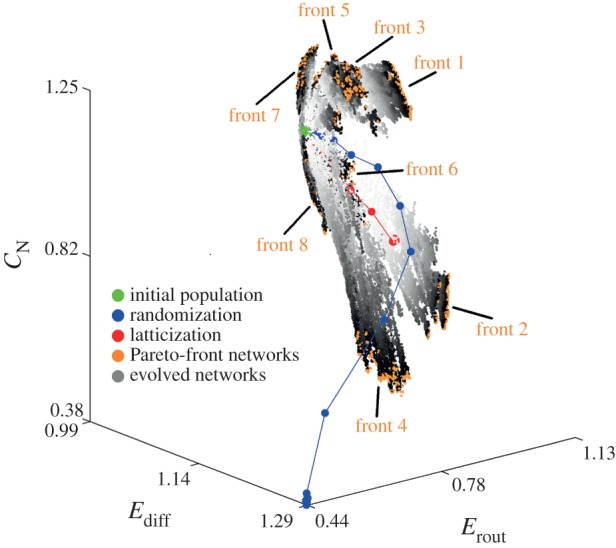
Efficiency-complexity morphospace of brain networks. Grey and orange points indicate the regions of the morphospace that have been explored by evolving a population of 500 brain networks. Networks are evolved employing a multi-objective optimization algorithm with eight distinct objective functions that drive networks towards eight quadrants of the morphospace. All objective functions impose distinct selective pressures over an evolving population of brain-like networks, resulting in eight final populations (orange points), called fronts, with distinctive structural properties. The greyscale assigned to each network indicates the epoch in which it was created, with light grey corresponding to early epochs and darker grey to later epochs. Blue and red points show the average trajectory of a randomized and latticized brain network, respectively, which are not subjected to any selective pressures. The spatial distribution of explored regions indicates that the accessibility of the morphospace is severely restricted: there are no networks found in the region *E*_diff_ < 1. Adapted with permission from reference [73].

An exploratory strategy similar to the one used in [72] was employed to perform a local exploration of a three-dimensional efficiency-complexity morphospace surrounding empirical brain networks (figure 6) [73]. The morphospace axes were defined by *E*_diff_ and *E*_rout_ (as defined in references [72]), and neural complexity (*C*_N_) [77], a measure of dynamic complexity that captures the coexistence of functional segregation and functional integration in neural systems. The exploration of this morphospace consisted of evolving a population of empirical brain networks towards eight octants of the three-dimensional morphospace. The exploration strategy was designed to take into account the limits in spatial volume and metabolic energy consumption that the brain is subjected to as a result of being a spatially embedded system (i.e. intrinsic constraints). Therefore, to ensure that all brain-like networks generated during the exploration belong to the FPT region and are biologically feasible, functionality and intrinsic constraints were imposed through a rewiring algorithm that preserves the total wiring volume of the network connections, the degree sequence and the connectedness of the networks. The explored morphospace of brain-like networks indicates that the accessibility to distinct regions is severely restricted; in fact, empirical brain networks are located at a minimum of *E*_diff_, where a boundary between possible and impossible brain-like topologies is defined.

While the tools to perform a deeper functional analysis of brain network morphospaces are still being developed, it is this kind of analysis that may open a door into the understanding of the underlying rules and constraints pervading the organization of brain networks. It is worth mentioning that the exploratory strategies used in [72,73] are not exhaustive; furthermore, we must emphasize that the accessibility of the morphospace is dependent on the region in which the exploration is initialized. Nonetheless, using Pareto-optimality as a selection criterion facilitates morphospace exploration, broadening the search directions and imposing fewer constraints on the search process. Its power to explore the morphospace resides in that it does not enforce network evolution to approximate a particular point within the morphospace nor does it require the definition of a global objective function. Pareto-optimality is domain-independent because it does not combine multiple objective functions into a single global metric; instead, it determines whether a solution can improve any of the objectives or not, regardless of the metric used for each objective. Hence, the multi-objective optimization is parameter-free and explores all possible trade-offs between objectives [76].

### 3.4. Network growth models

Defining morphospace axes as the parameters of a network growth model vastly simplifies the exploration of the space. However, there are a couple of considerations that must be taken into account during the morphospace analysis. First, as it was pointed out previously, for every set of parameter values, it is necessary to generate several instances of the model (unless the model is deterministic). Then, the question is, how to assign a representative topology to every point in the morphospace, given that there are several different networks associated with each point in the space. The answer to this question depends on the degeneracy of the parameters; that is, how much variability exists in an ensemble of networks generated by the same set of parameter values. On the one hand, if a set of parameter values are highly degenerate, it might be more appropriate to consider the distribution of a set of structural measures, evaluated over the ensemble of networks. On the other hand, if there are no significant degeneracies in the parameters, then one can either average over all networks or choose a representative network such that its similarity to all other networks is maximal.

**Figure 7.**
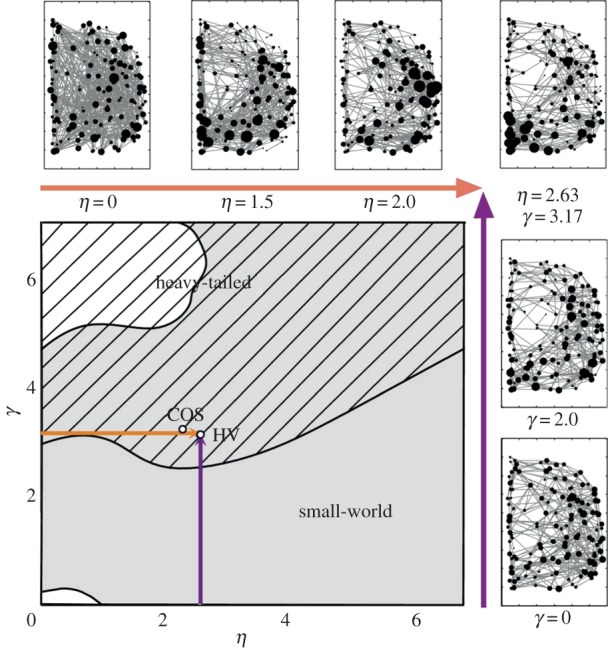
Brain network morphospace. Axes of the morphospace are given by the parameters *γ* and *η* of the network growth model 
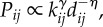
 where *P_ij_* is the probability that a functional connection exists between nodes *i* and *j*; *k_ij_* is the number of nearest neighbours that nodes *i* and *j* have in common; *d_ij_* is the spatial distance between nodes *i* and *j.* Distinct combinations of parameter values generate specific network topologies, which are indicated by the different shaded regions in the plot. The parameters values that best capture the topological features of healthy brain networks (HV) and of participants with childhood-onset schizophrenia (COS) are indicated with white dots within the region containing networks with heavy-tailed degree distributions. Adapted with permission from reference [78].

A second question that comes up when using growth models is how to locate real-world networks within the morphospace. In other words, given a growth model, how does one determine the parameter values that correspond to an empirical network? Vértes *et al*. [78] used simulated annealing in various two-dimensional morphospaces in order to estimate model parameters that best capture the topological features observed in anatomically embedded brain networks. Three different models were suggested to determine the probability *P_ij_* of a functional connection between pairs of cortical regions. The best fitting model consisted of two competing terms, one penalizing costly connections, and a second term favouring nodes that share nearest neighbours; each term was associated with a parameter that regulates its importance in determining the probabilities *P_ij_*. Simulated annealing was used on an energy function that measures the similarity between a network generated by the model and empirical brain networks (figure 7).

In general, to estimate the parameters that best fit an empirical network, any optimization process over an energy (similarity) function may be used. Similar to parameter degeneracies, the energy landscapes can also suffer from such degeneracies when two or more networks have equal energy values (i.e. they are equally similar to the empirical network), but exhibit significantly different topologies. However, the use of generative models for morphospace analysis has the power to shed light on the processes driving network formation. Furthermore, an interesting and more informative approach might be to use growth models in combination with morphospaces whose axes are defined independently by structural traits. Esteve-Altava *et al.* [79,80] have given a first step in this direction by modelling tetrapod skulls as networks of bones connected by sutures. In reference [80], four skull network morphospaces are defined by two structural (morphological) traits, namely number of nodes (bones) and number of connections (sutures). The four morphospaces were explored independently, according to four growth models that were used to generate networks in each morphospace. Each growth model generated networks in distinctive regions of the morphospace, depending on the parameter values of the model. For instance, parameter values that produce disconnected networks, loops (self-connections) or redundant connections define a restricted region of functionally unviable skull topologies. Finally, by mapping a set of empirical skull networks onto the morphospaces the authors show that a *proximal constraint* model [81] can better fit the empirical data. Therefore, the authors conclude that this model is able to explain the possible developmental processes underlying the formation and evolution of tetrapod skull, in which bones establish suture connections according to their pairwise geometric distances.

The methodology used in reference [80] is interesting in that (i) it provides a systematic mechanism to explore a morphospace whose axes are defined by structural traits; (ii) the exploratory processes highlight biologically plausible regions (within the FPT regions), and enable the authors to test different hypothesis about the underlying evolutionary and developmental processes driving skull formation. Regarding the analysis provided by this paper, there are two observations worth noting. First, the *proximity constraint* model that provides the better fit of the empirical data is also, among the four models tested, the only one that takes geometrical and spatial considerations into account. Both these features are crucial in the organization of any spatially embedded network, such as skull networks. Therefore, it is not surprising that this model yields a closer fit. Second, while the *proximity constraint* model generates networks within a narrow, constrained region of the morphospace that includes all empirical networks, it should be noted that this assessment is only based on two structural traits (number of nodes and connections) and that other topological characteristics of the generated networks have not been investigated and compared with the empirical networks. Hence, a more detailed investigation of the morphospaces is required in order to assess the viability of a generative model as a hypothesis for evolutionary and/or developmental processes.

Using growth models in combination with optimization methods to explore morphospaces whose axes are defined by structural traits could be a more exhaustive method to uncover distinct morphospace regions. As far as we know, this kind of work has not been done previously, but could be a promising future direction to apply network morphospace analysis to the study of real-world networks.

## 4. Concluding remarks

This paper reviews the theoretical foundations and a number of recent applications of the concept of ‘network morphospace’, based on ideas that were derived from the concept of theoretical morphospace used in the study of the evolution of organismic morphology. Network theoretical morphospaces are a useful tool for classifying and mapping network architectures according to a set of structural characteristics. While there are a number of different approaches to try to identify and classify key features of network structure, we have seen that some of these approaches can be reformulated in terms of a morphospace analysis. In addition, such morphospace analysis does not only lead to a network classification, but it also provides information about the possible factors driving the organization of network structure, how certain structural features emerge and how such features interact with each other.

It is worth noting that network morphospaces provide an operational framework to study the evolution of interactions in complex systems in a computational setting, through the application of selection pressures that push systems along one or several competing directions. In this way, in addition to finding the place of our systems within the morphospace, we are able to test how likely their presence is in particular domains and ultimately how evolvable they are. Finally, the most important application of morphospaces is that they offer a framework to analyse real-world networks and try to answer one of the most difficult questions of evolution [82]: ‘why do networks look the way they do and not any other way’?
